# Hyperbaric oxygen upregulates cochlear constitutive nitric oxide synthase

**DOI:** 10.1186/1471-2202-12-21

**Published:** 2011-02-22

**Authors:** Chia-Der Lin, I-Hua Wei, Chih-Ho Lai, Te-Chun Hsia, Ming-Ching Kao, Ming-Hsui Tsai, Ching-Hsiang Wu, Mang-Hung Tsai

**Affiliations:** 1 Department of Otolaryngology-Head and Neck Surgery, China Medical University Hospital, Taichung, Taiwan; 2 Graduate Institute of Clinical Medical Science, School of Medicine, China Medical University, Taichung, Taiwan; 3 Department of Anatomy, School of Medicine, China Medical University, Taichung, Taiwan; 4 Department of Microbiology, School of Medicine, China Medical University, Taichung, Taiwan; 5 Hyperbaric Oxygen Therapy Center, China Medical University Hospital, Taichung, Taiwan; 6 Department of Biological Science and Technology, College of Life Sciences, China Medical University, Taichung, Taiwan; 7 Department of Biochemistry, National Defense Medical Center, Taipei, Taiwan; 8 Department of Biology and Anatomy, National Defense Medical Center, Taipei, Taiwan

## Abstract

**Background:**

Hyperbaric oxygen therapy (HBOT) is a known adjuvant for treating ischemia-related inner ear diseases. Controversies still exist in the role of HBOT in cochlear diseases. Few studies to date have investigated the cellular changes that occur in inner ears after HBOT. Nitric oxide, which is synthesized by nitric oxide synthase (NOS), is an important signaling molecule in cochlear physiology and pathology. Here we investigated the effects of hyperbaric oxygen on eardrum morphology, cochlear function and expression of NOS isoforms in cochlear substructures after repetitive HBOT in guinea pigs.

**Results:**

Minor changes in the eardrum were observed after repetitive HBOT, which did not result in a significant hearing threshold shift by tone burst auditory brainstem responses. A differential effect of HBOT on the expression of NOS isoforms was identified. Upregulation of constitutive NOS (nNOS and eNOS) was found in the substructures of the cochlea after HBOT, but inducible NOS was not found in normal or HBOT animals, as shown by immunohistochemistry. There was no obvious DNA fragmentation present in this HBOT animal model.

**Conclusions:**

The present evidence indicates that the customary HBOT protocol may increase constitutive NOS expression but such upregulation did not cause cell death in the treated cochlea. The cochlear morphology and auditory function are consequently not changed through the protocol.

## Background

Hyperbaric oxygen therapy (HBOT) is an effective treatment for decompression Sickness (DCS) and arterial gas embolism (AGE)[1], and is proposed as an adjunct in treating ischemia-related inner ear diseases [2-4], sudden deafness [4,5], and acute noise trauma [6,7]. The efficacy of HBOT in treating these inner ear diseases is variable, and the mechanism of HBOT in inner ears is still not fully understood. For example, HBOT has been proposed to be an effective rescue strategy for noise trauma [6,7]; however, one study found that HBOT had an adverse effect on the inner ear after noise trauma [8]. Consequently, a high degree of medical skepticism still exists regarding the role of HBOT in treating inner ear diseases. More studies on the functional and cellular changes that occur after HBOT may help to elucidate these conflicting results.

Nitric oxide (NO) has important roles in cochlear physiology, including neurotransmission [9], regulation of cochlear blood flow [10,11], homeostasis of cochlear endolymph [11] and induction of cytotoxicity under pathological conditions [12,13]. NO is synthesized by nitric oxide synthase (NOS) with the conversion of L-arginine to L-citrulline. Three isoforms of homologous NOS have been identified, including two constitutive isoforms (neuronal NOS [nNOS or NOS I] and endothelial NOS [eNOS or NOS III]) and one inducible isoform (iNOS or NOS II) [11,14,15]. Constitutive NOS is calcium/calmodulin dependent and is continuously expressed, whereas iNOS is calcium independent and is expressed in response to specific stimulants such as cytokines or endotoxins [12]. Constitutive isoforms of NOS, both nNOS and eNOS, are expressed in the normal cochlea [16], but iNOS is expressed in the cochlea only after exposure to some pathologic conditions such as endotoxins [12], ischemia [13] or acoustic trauma [17]. The aim of this study was to investigate the effects of repetitive HBOT on cochlear function and the expression of NOS isoforms by means of immunohistochemical staining.

## Results

### No significant auditory changes after repetitive customary HBOT

**Figure 1:**
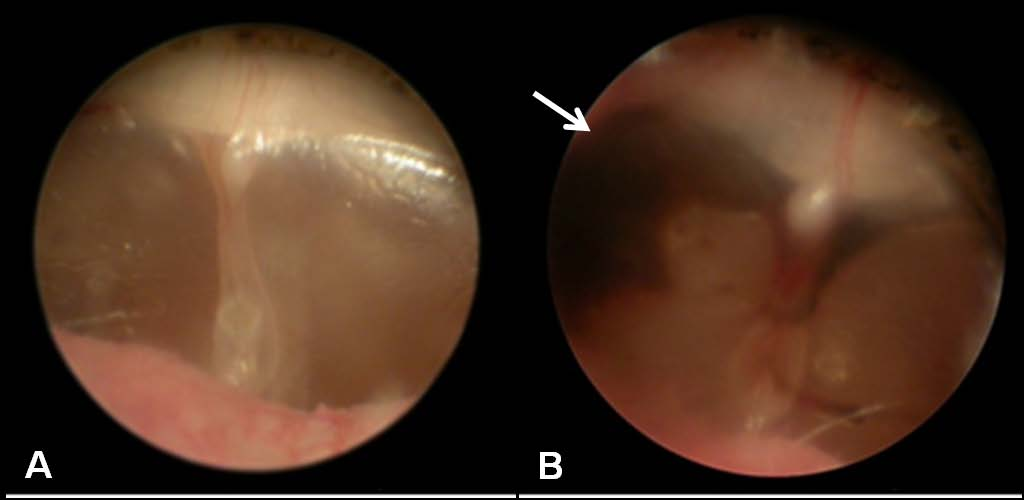
**Morphology of eardrum after HBOT**. Otoscopic view of the eardrums in guinea pigs. A. normal eardrum; B. Image of the eardrum with moderate hemorrhage (arrow) after 20 sessions of HBOT.

All the animals in the experimental group tolerated the entire course of HBOT without signs of irritability or discomfort. All eardrums were checked and were found to be normal before the study. After 20 HBOT sessions, one ear still remained clean and normal without evidence of hemorrhage (Teed grade 0), one ear showed slight vascular injection (Teed grade 1), seven ears developed minor hemorrhage (Teed grade 2) and one ear developed moderate hemorrhage in the eardrum (Teed grade 3; Figure 1). No hemotympanum or eardrum perforation was observed in the experimental group. The eardrum condition in the control group remained normal throughout the duration of this study.

**Figure 2:**
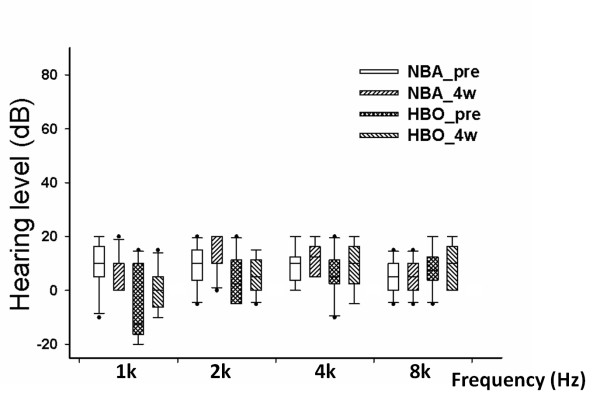
**Auditory level**. Box plots of the hearing level in the control, normobaric air (NBA) group and the experimental, hyperbaric oxygen (HBO) treatment group before (_pre) and 4 weeks after (_4w) the treatment sessions. There were no significant intra-group changes or inter-group differences in the hearing levels before and 4 weeks after the start of the treatment. *X*-axis indicates the different frequencies for auditory measurement, from 1 k to 8 k Hz. The dots represent the outliers.

Tone burst auditory brainstem responses (ABRs) were used to assess hearing before and after HBOT (Figure 2). In the control normobaric air (NBA) and experimental HBOT groups, the intragroup hearing level prior to the study and four weeks after commencing the study did not significantly differ. Although slight elevated hearing level at 1 kHz was recorded in the NBA group, the intergroup hearing level between the control NBA and experimental HBOT groups did not significantly differ (Figure 2).

### Differential expression of NOS isoforms in different cochlear regions after repetitive HBOT

**Figure 3:**
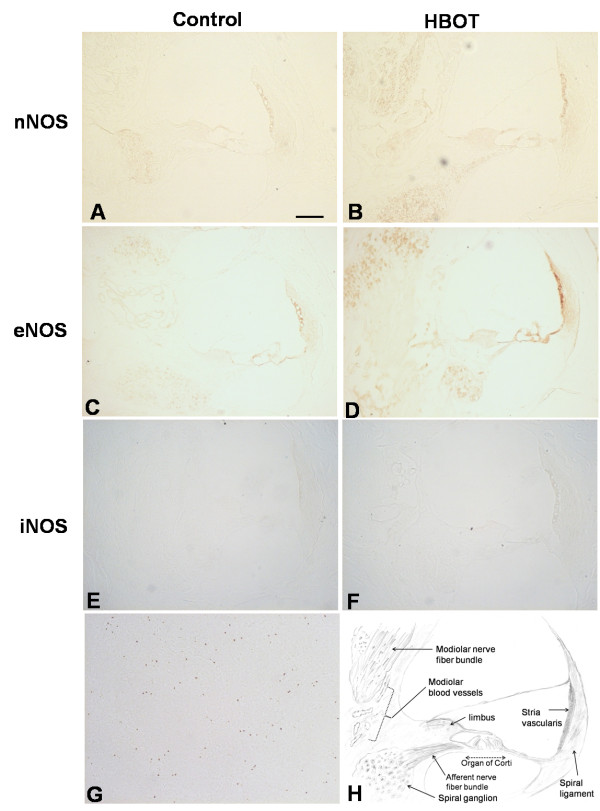
**Immunohistochemistry of NOS subtypes in cochlea**. Immunohistochemistry of cochlear nNOS (A,B), eNOS (C,D) and iNOS (E,F) expression in the control NBA and HBOT groups. (G) Spleen tissue was used as a positive control for iNOS. (H) Schematic drawing depicting a cross-section through cochlear canals and its related sub-structures. (A,B) nNOS immunoreactivity was present in the spiral ganglion, stria vascularis, organ of Corti, limbus and modiolar nerve bundles in both groups but enhanced immunoreactivity was present in the HBOT group. (C,D) eNOS immunoreactivity was found in the modiolar blood vessels, stria vascularis, organ of Corti, limbus and spiral ganglion in both groups with more enhanced immunoreactivity in the HBOT group. iNOS did not exhibit immunoreactivity in the control NBA (E) and HBOT (F) groups, as compared with the positive control of iNOS expression in the spleen tissue (G). Scale bar, 50 μm.

**Figure 4:**
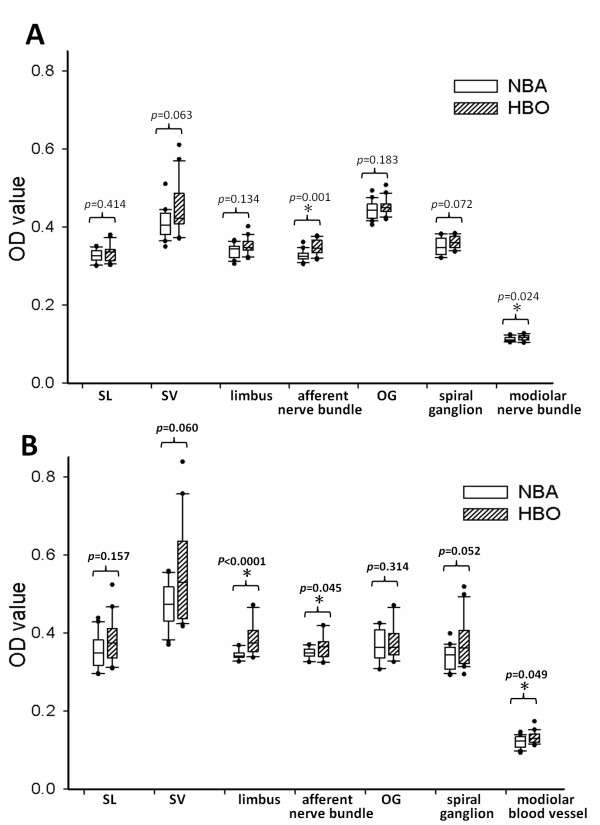
**Morphometric analysis of nNOS and eNOS**. Box plots of the optical densities from morphometric measurements of nNOS (A) and eNOS (B) in the control NBA (in white box) and experimental HBO treatment group (in dash box). The expression of nNOS was significantly enhanced in nerve fiber bundles after HBOT. The expression of eNOS was significantly enhanced in the limbus, nerve fiber bundles and modiolar blood vessels after HBOT. The dots represent the outliers. OD, optical densities; SL, spiral ligament; SV, stria vascularis; OG, organ of Corti.

**Figure 5:**
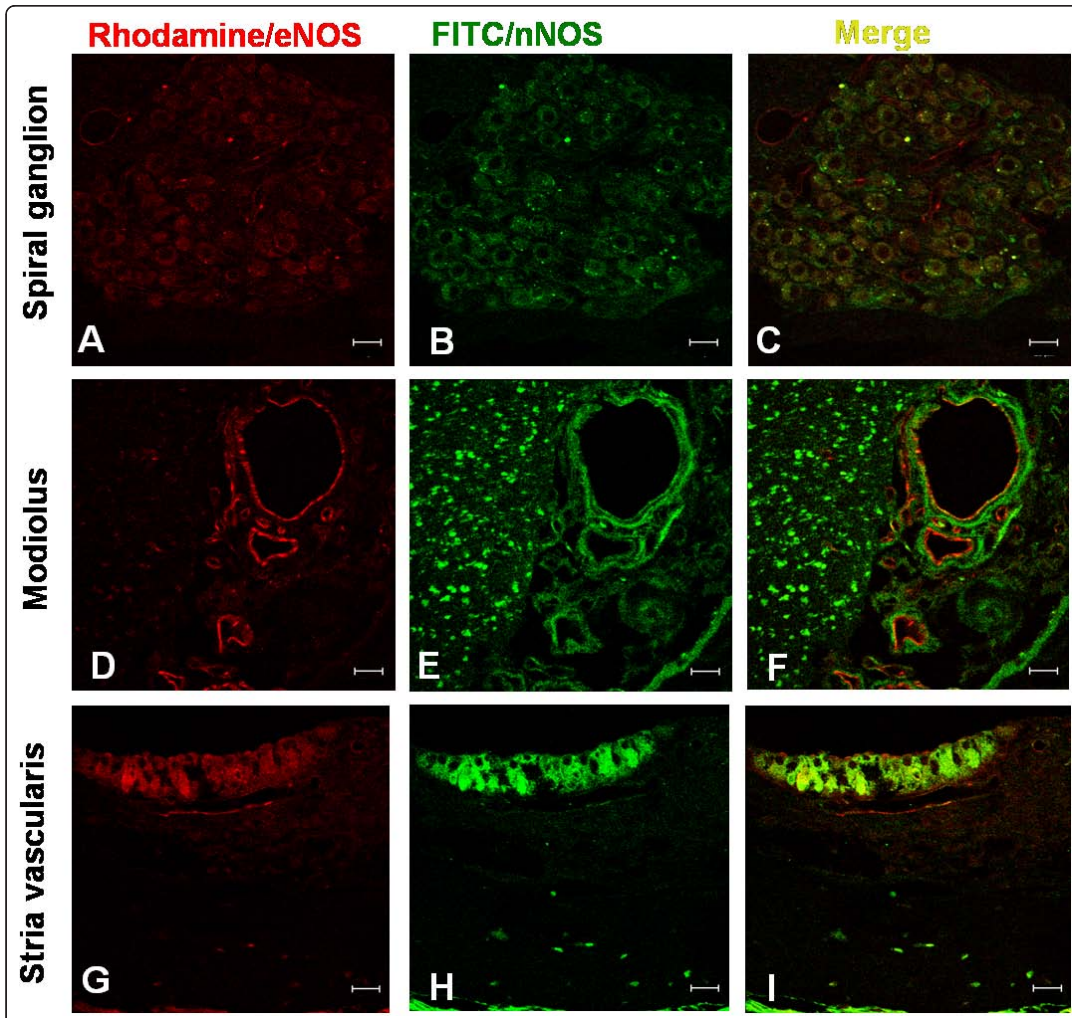
**Confocal microscopic view of eNOS and nNOS after HBOT**. Immunofluorescence labeling of eNOS (red) and nNOS (green) in the spiral ganglion (A-C), cochlear modiolus (D-F) and stria vascularis (G-I) of the cochlea after repetitive HBOT. Co-expression of eNOS and nNOS occurred in the spiral ganglion (C) and stria vascularis (I). In the cochlear modiolus, nNOS was expressed in the modiolar nerve fibers, and eNOS was expressed along the modiolar blood vessel. Scale bar, 20 μm.

The immunohistochemical results for NOS expression in cochleae from the control group and the experimental group are illustrated in Figures 3, 4 and 5. In the control NBA group, nNOS exhibited moderate immunoreactivity in the spiral ganglion and stria vascularis and faint immunoreactivity in the modiolar nerve fibers, afferent nerve fibers, limbus and organ of Corti (Figure 3A). In the experimental HBOT group, enhanced immunoreactivity of nNOS was present in the spiral ganglion, modiolar nerve fibers, afferent nerve fibers and stria vascularis (Figure 3B). Using semi-quantitative morphometric analysis, significant enhanced immunoreactivity of nNOS was noted in the nerve fiber bundles in the HBOT group (Figure 4A).

Immunohistochemistry using the antibody against eNOS in the control NBA group revealed moderate immunoreactivity in the stria vascularis, organ of Corti, limbus, spiral ganglion and modiolar blood vessels (Figure 3C). In the experimental HBOT group, enhanced immunoreactivity of eNOS was present in the spiral ganglion, modiolar blood vessels, limbus and stria vascularis (Figure 3D). Significantly enhanced eNOS immunoreactivity was noted in the modiolar blood vessels, nerve fibers and the limbus in the HBOT group (Figure 4B).

Immunohistochemistry using the antibody against iNOS did not reveal immunoreactivity in the NBA and HBOT groups, as compared with the iNOS expression in the positive control spleen tissue (Figures 3E, 3F and 3G).

To assess the co-expression of nNOS and eNOS in the cochlea after HBOT, we used confocal microscopy (Figure 5). Co-expression of nNOS and eNOS was noted in the spiral ganglion and stria vascularis. Single expression of eNOS immunoreactivity was also present in the capillaries within the spiral ganglion region (Figure 5). In the cochlear modiolus, nNOS was expressed in the modiolar nerve fibers, whereas eNOS was expressed along the modiolar blood vessels.

### No obvious DNA fragmentation after HBOT by TUNEL assay

**Figure 6:**
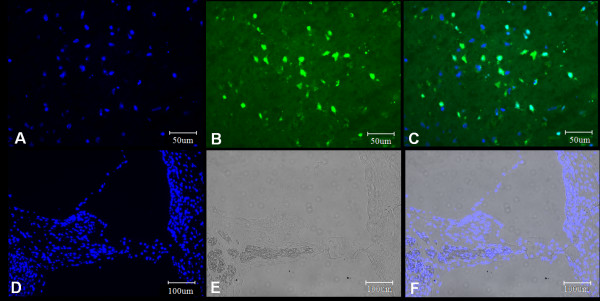
**TUNEL assay of cochlea after HBOT**. TUNEL assay of cochleae after HBOT. Significant DNA fragmentation was shown in the brain tissue with penetrating injury (A-C); however, no evidence of DNA damage was identified in the cochlear tissue after HBOT (D-F).

To determine if there was any DNA fragmentation or apoptotic cell death after HBOT, we used the TUNEL assay to evaluate the cellular changes after HBOT. DNA fragmentation and possible apoptotic cell death were observed in the positive control obtained from brain tissue with penetrating damage (Figures 6A, 6B and 6C); however, no DNA fragmentation was found in the cochlear tissue after HBOT (Figures 6D, 6E and 6F).

## Discussion

Our present study, which was designed according to the HBOT protocol used in clinical practice for acute cerebral ischemia [18], provides a functional measurement and immunohistochemical evidence of cochlear NOS changes as well as morphological evaluation of the eardrum after HBOT. HBOT effects on hearing may be caused by decompression illnesses or barotraumas to the middle or inner ear [19]. A slight increase in hearing sensitivity is evident in animals receiving extreme acute hyperbaric conditions [20]. The increased hearing sensitivity may be caused by changes in the tympanic membrane and middle ear impedance, which may be altered under hyperbaric conditions [20]. In this study, the tympanic membrane and mesotympanum were observed by otoscopy after repetitive HBOT. No significant changes in tympanic membrane or mesotympanic injury were observed, with the exception of one ear with moderate hemotympanum (Figure 1). Because of their widely patent Eustachian tube, guinea pigs have been shown to tolerate compression well, with minimal middle/inner ear barotrauma even under rapid compression rates such as 2 ATA in 2 seconds [21]. Any barotrauma due to repetitive HBOT might gradually improve without significant sequelae [22]. Therefore, the hearing threshold was not significantly affected by the minor changes in the eardrum and middle ear (Figure 2).

Transient deterioration of cochlear function during the initial exposure to HBOT has been observed in guinea pigs and rabbits, which has been attributed to the vasoconstriction of cochlear blood vessels during the initial exposure to hyperbaric oxygen [23]. Hyperbaric oxygen causes a slight morphologic alteration in the outer hair cells of newborn rats [24]. After repetitive HBOT, minor changes in cochlear function were observed including cochlear degeneration, inner hair cell damage and hemorrhage in the perilymphatic space and scala media [25]. The cochlear hemorrhage phenomenon after hyperbaric treatment was also reported in rats [26]. The cochlear changes observed in these studies [25,26] might be caused by higher-pressure conditions and a more-rapid compression-decompression process, since cochlear degeneration and hemorrhage were more pronounced under higher-pressure conditions (up to 5 ATA) [25]. In that study, the rate of compression was >0.3 ATA/min, and decompression was about 0.5 ATA/min. These conditions exceed the standard HBOT protocols used in clinical practice. With slower compression-decompression procedures and more modest maximum compression pressures, no significant changes in hearing levels were observed after repetitive HBOT [22,27]. In this study, a slower compression-decompression protocol (≤0.2 ATA/min) and lower peak pressure (2.5 ATA) more closely mimic the current HBOT protocol used in clinical practice. It is of note that no significant shifts in the hearing level or cell death by TUNEL assay were observed before and after HBOT using this animal model. We suggest the results of this study more accurately reflect the clinical effects of HBOT on the cochlea.

The elevation of the hydrostatic pressure while breathing oxygen during HBOT will increase the partial oxygen in target tissues providing the animal has a functioning cardiorespiratory system and the target tissue is perfused. After hyperbaric treatment at 2.5 ATA with pure oxygen, partial oxygen pressure in the cochlear perilymph may increase by up to 5-fold [2]. By this mechanism, HBOT may be effective for sudden sensorineural hearing loss where ischemia may be the cause [4,5]. This may also include noise-induced hearing loss [6,7]. In addition to elevation of partial oxygen pressure, oxidative stress is believed to be fundamental to the therapeutic mechanisms for HBOT [28]. Reactive nitrogen species, including NO, contribute to this stress [28]. NO is synthesized by different NOS isoforms and the expression of these isoforms varies both from tissue to tissue and with the application of HBOT. We might therefore expect a range of effects from HBOT in different target tissues. The cerebrovascular responses to hyperoxia may be modulated by eNOS and nNOS-derived NO [29]. Hyperbaric oxygen exposure may enhance sensitivity to seizure because of the early cerebral vasoconstriction by eNOS-derived NO [30]. Repetitive HBO exposure may further elevate NO content in the brain, which may promote an imbalance between glutamatergic and GABAergic synaptic function and the genesis of oxygen-induced seizures [31]. In contrast, HBOT preconditioning may protect myocardium from subsequent ischemia/hypoxia by way of upregulating eNOS [32]. Therefore, HBO may be beneficial or harmful depending on the type of tissue.

Different NOS isoforms are expressed in discrete regions of the cochlea. The constitutive NOS, nNOS and eNOS, are distributed in the substructures of the cochlea under normal physiologic conditions [16]. nNOS immunoreactivity is found in the hair cells, spiral ganglion, stria vascularis, spiral ligaments, limbus and nerve fibers and spiral ganglion, whereas eNOS immunoreactivity is present in the endothelium of the cochlear microvascular trees, stria vascularis, limbus and the spiral ganglion [14,16]. iNOS was not found in the structures of the normal cochlea [15]. iNOS is expressed in the cochlea only after exposure to some pathologic condition such as endotoxin [12], ischemia [13] or acoustic trauma [16,17]. In this study, the distribution of nNOS and eNOS was similar to that of previous studies [14,16]. nNOS was distributed in the spiral ganglion, nerve fiber bundles, stria vascularis, limbus and hair cells. eNOS was distributed along the microvascular structures in the cochlea such as the endothelium of blood vessels in the modiolus, stria vascularis, spiral ganglion and limbus. After repetitive HBOT, constitutive NOS immunoreactivity was enhanced in these regions (Figure 3). Morphometric analysis depicted the significantly enhanced immunoreactivity of nNOS in nerve fiber bundles and that of eNOS in nerve fiber bundles, limbus and modiolar blood vessels (Figure 4). Marginally significant enhanced immunoreactivity was demonstrated in stria vascularis and spiral ganglion. Co-expression of eNOS and nNOS was also shown in stria vascularis and spiral ganglion after HBOT (Figure 5). These indicate that the possible therapeutic role of HBOT may be through the upregulation of constitutive NOS in the cochlear neurovascular substructures since mild elevation of NO may provide a neuroprotective effect on auditory hair cells under ischemic condition [15]. We have 10 cochleae for each group in this study. Regarding the marginal significance in some effects such as spiral ganglion and stria vascularis, our study was underpowered to determine the statistical significance of such effect. For example, given the sample size and effect size we had in the current study, the power for the immunoreactivity (reflected as the optical densities, [OD]) of eNOS comparison between HBOT and NBA group for spiral ganglion was 0.47. Future studies will need to include larger numbers of animals in order to confirm or refute these marginal findings.

To our knowledge, this is the first report of the upregulation of constitutive NOS in some substructures of the cochlea after HBOT treatment. Constitutive NOS may act as a protective enzyme. In cerebrovascular tissue, hyperbaric oxygen can elicit a vasorelaxing effect from constitutively active NO by eNOS and nNOS [29], although eNOS-derived NO may have some vasoconstrictive effect during the early exposure to HBO [30]. NOS produces NO, which induces relaxation of the smooth musculature and regulation of the vasotonia as a microbiotic messenger [29]. Thus, the important role of NO in cardioprotection [32] and cerebroprotection [33] against ischemic damage is established. NO released from constitutive NOS such as eNOS also protects cochlear venules from excessive venular leakage [34]. In contrast, iNOS usually has a devastating role in biological processes. Induction of iNOS has also been demonstrated in some cochlear pathologies like ischemia [13] or noise trauma [17]. When the cochlea is exposed to hypoxic or ischemic conditions, the expressed iNOS may lead to an overexpression of peroxides [30], which subsequently induce a direct toxic effect on neurons and may affect the endocochlear potential. The synaptic complex between the hair cells and the nerve fibers is another region that the NO may exert its role [35]. NO could inhibit the glutamate receptors by positive feedback under normoxic conditions. Conditions causing cochlear hypoxia such as acoustic overstimulation may induce glutamate release and calcium influx at the synaptic complex between the hair cells and the nerve fibers [35] and increase iNOS expression with excessive formation of NO [17]. Upon glutamate release, overproduction of iNOS-derived NO can further increase the cochlear oxidative stress and cochlear dysfunction. In this study, only constitutive NOS, especially eNOS, was upregulated after HBOT, whereas iNOS was not immunoreactive. Thus, customary HBOT may play a therapeutic role rather than an adverse affect on cochlear pathology.

## Conclusion

This study showed a differential effect of HBOT on the expression of NOS isoforms. Upregulation of constitutive NOS was shown in the substructure of the cochlea after HBOT, but iNOS was not expressed. Such observations should be extrapolated to the humans with caution, but support a therapeutic role for HBOT. There were no significant changes in the eardrums or ABR threshold shifts, and there was no obvious DNA fragmentation after HBOT in the guinea pig model.

## Methods

### Animals

Adult male albino guinea pigs (400-600 g, 10-14 weeks of age) with intact Preyer reflexes and normal eardrums were used in this study. Animals were housed in groups under diurnal lighting conditions, and regular guinea pig diet and water were provided *ad libitum*. Animal use protocols were approved by the China Medical University Committee on Use and Care of Animals (permission number: 96-75-N). The experimental HBOT group included five animals that received regular repetitive HBOT (see below). The control, normobaric air (NBA) group included five animals that did not receive HBOT and were maintained in normobaric room air.

### HBOT Model

Hyperbaric oxygen experiments were conducted in a balloon-bag acrylic chamber inside a large pressure chamber (Hyperbaric Oxygen Center in the China Medical University Hospital). The HBOT protocol used in the study was similar to the HBOT protocol used in clinical practice for acute cerebral ischemia [18]. The temperature inside was maintained between 22 and 26°C with relative humidity at ~60%. The chamber pressure was steadily increased to a pressure of 2.5 atmosphere absolute (atm abs. or ATA). Compression and decompression were carried out at a rate of 0.2 ATA/min. Each treatment consisted of 17 min of compression time, 60 min of stable compression time at 2.5 ATA and 13 min of decompression time. The animals were continuously observed during the course of HBOT to manually adjust the oxygen ventilation rate and observe the behavior of the animals, particularly for signs of irritability or discomfort. A balloon that indicates the pressure balance between the inside and outside of the small chamber was mounted on the side wall of the small acrylic chamber. The animals in the experimental group received 20 HBOT treatments over a 4-week period (once per weekday, five times per week).

### Otoscopic Evaluation

To minimize the frequency of anesthesia, visual assessment of the tympanic membrane was performed associated with the procedure for auditory brainstem response (ABR) measurements (described below). After guinea pigs were anesthetized with intramuscular injection of zoletil (30 mg/kg) and xylazine (10 mg/kg), photographs of the tympanic membrane were obtained using a Storz tele-otoscope. The severity of barotrauma on the tympanic membrane was graded using a modified Teed classification scheme [36]: 0, normal; 1, slight vascular injection or retraction of the eardrum; 2, mild hemorrhage in the eardrum; 3, gross hemorrhage in the eardrum; 4, hemotympanum; 5, tympanic membrane perforation.

### Auditory Test

Hearing tests were performed by tone burst ABR in a sound-attenuated room, before and after completion of the 20 HBOTs. The pure tone bursts were generated with the amplitude specified by a real-time programmable attenuator (Intelligent Hearing Systems, IHC Smart EP version 3.97, Miami, FL, USA) with ER2 insert earphone, with stimulus frequencies of 1, 2, 4 and 8 kHz (0.2-ms rise/fall time and 1-ms flat segment) with maximal output levels of 125, 123, 111 and 117 dB sound pressure level (SPL). The tone bursts were produced by an IHS transducer (IHS Inc., Miami, FL, USA) in a closed acoustic system through the sound delivery system. Responses for 1024 sweeps were averaged at each intensity level around the threshold in 5-dB SPL steps. Threshold was defined as the lowest intensity level at which a clear waveform was visible in the evoked trace and was determined by visual inspection of the responses. At least two sequences of recordings were made at the threshold intensity to verify the reproducibility of the ABR responses. ABR threshold at each time and at each frequency was compared with the pre-surgical threshold as a baseline. Threshold shift values were estimated.

### Immunohistochemistry

Animals were sacrificed after the 20 HBOT treatments. They were first anesthetized by intramuscular injection of zoletil (30 mg/kg) and xylazine (10 mg/kg) and then perfused intracardially with 4% paraformaldehyde in 0.1 M phosphate-buffered solution (PBS) at pH 7.4. The temporal bones and spleen tissues of the animals were removed. The cochleae were opened at the apex and round window and oval window membranes for better penetration of the paraformaldehyde. The temporal bones were immersed in the same fixative overnight at 4°C. Decalcification was performed with 0.1 M EDTA solution, buffered with PBS to pH 7.4, for 4 weeks at 4°C. Serial sections (7 μm thick) were cut using a microtome in a plane parallel to the long axis of the cochlea and mounted on silane-coated slides for further immunohistochemical analysis. At least six sections obtained from the modiolus in each animal were immunostained. Serial sections (7 μm thick) of spleen tissue were also cut using a microtome and mounted on silane-coated slides for further immunohistochemical analysis to act as positive control for the expression of iNOS.

A standard avidin-biotin-peroxidase (ABC) method was used to locate NOS immunoreactive regions [37]. In brief, the sections were rinsed with 0.05 M Tris-buffered solution (TBS) at pH 7.4 and then incubated in 3% H_2_O_2 _for 1 h, followed by 0.1% Triton X-100 in 10% serum (normal goat serum [NGS] for eNOS/iNOS and normal horse serum [NHS] for nNOS) for 1 h. Subsequently, samples were incubated overnight at 4°C with the primary antibodies to nNOS (mouse monoclonal, 1:100, Santa Cruz Biotechnology, Santa Cruz, CA, USA), eNOS (rabbit polyclonal, 1:1000, Santa Cruz Biotechnology) or iNOS (rabbit polyclonal, 1:2000, Santa Cruz Biotechnology). The sections were then incubated with the secondary antibodies, either biotin-conjugated goat anti-rabbit IgG for eNOS and iNOS or anti-mouse IgG for nNOS (Sigma, St. Louis, MO, USA), diluted 1:200 in 2% serum (NGS for eNOS and iNOS and NHS for nNOS). The reaction was developed with a horseradish peroxidase-streptavidin complex (Dako A/S, Denmark) at a 1:300 dilution for 1 h, followed by 0.06% 3,3'-diaminobenzidine (DAB, Sigma) with 0.066% H_2_O_2 _substrate medium in 0.05 M TBS. All specimens were then dehydrated in a graded series of ethanol and embedded in a Clearmount mounting solution (Zymed, USA). Sections from each of the experimental animals were immunostained during the same run to allow comparisons across the groups. The spleen from each animal was subjected to the same fixation process, sectioned and immunostained to serve as the iNOS-positive control. Sections were photographed and analyzed using a Zeiss Axioskopz light microscope (Axioskop 2, Zeiss, Germany).

### Semi-quantitative morphometric analysis

The intensities of nNOS and eNOS immunoreactivity were measured in the cochlea by comparing the optical densities (ODs) of the immunoreactivity in the control (normal room air, NBA) and experimental (hyperbaric oxygen treatment, HBOT) groups using the image analyzer (Image Pro Plus III, Media Cybernetics, USA) [38]. Two comparative paramodiolar sections were sampled and analyzed from the apical to the basal turn of the cochleae from both ears of each animal. Twenty different cochlear sections were analyzed in each group, including 80 different regions of each Corti's organ. The total areas of the selected immunoreactive regions were calculated and compared between the control and experimental groups. The packing ODs of the target regions in each cochlea were calculated and compared based on the summation of ODs relative to the selected areas in each cochlear turn, from the apical to basal region.

### Immunofluorescence labeling and confocal microscopy

To study the co-expression of nNOS and eNOS in the cochlear sections, immunofluorescence labeling of eNOS and nNOS was performed. The sections were rinsed with 0.05 M TBS at pH 7.4 and then incubated in 3% H_2_O_2 _for 1 h, followed by 0.1% Triton X-100/10% NGS in PBS for 1 h. Subsequently, they were incubated overnight at 4°C with the primary antibodies to nNOS (1:100 dilution, Santa Cruz Biotechnology) and eNOS (1:1000 dilution, Santa Cruz Biotechnology). The sections were then incubated with the secondary antibodies, FITC-conjugated goat anti-mouse IgG for nNOS and rhodamine-conjugated goat anti-rabbit IgG for eNOS (Sigma), diluted 1:200 by 2% NGS in PBS. Finally, the section slides were examined in a confocal laser scanning microscope (LSM510, Zeiss).

### *In situ *detection of nuclear DNA fragmentation

We used the terminal deoxynucleotidyl transferase (TdT)-mediated deoxyuridine triphosphate (dUTP)-biotin nick end labeling (TUNEL) method to detect DNA fragmentation. The TUNEL assay was performed using an *in situ *cell death detection kit with a fluorescein label (Roche Diagnostics GmbH, USA). The kit contains TdT, which catalyzes the polymerization of fluorescein dUTP to free 3'-OH DNA ends in a template-independent manner. TUNEL-positive cells were identified by incorporation of fluorescein-conjugated dUTP. According to the manufacturer's instructions, the sections were pre-treated with permeabilization solution (0.1% Triton X-100 in 0.1% sodium citrate) for 2 min on ice (4°C) and then incubated for 60 min at 37°C with the TUNEL reaction mixture. After washing in PBS, sections were photographed in a laser scanning confocal microscope (Zeiss). Brain tissue from guinea pigs that suffered penetrating trauma (under anesthesia) 3 days prior to sacrifice was used as a positive control [39].

### Statistical analysis

All values from groups of animals were presented in box plots, which included medians, 25^th ^percentile, 75^th ^percentile, minimum and maximum. For the comparison between groups, the nonparametric Mann-Whitney *U *test contained in the SPSS program (version 12.0 for Windows, SPSS Inc., Chicago, Illinois, USA) was applied. The differences were considered to be statistically significant when *p *< 0.05.

## Abbreviations used

ABR: auditory brainstem response; HBOT: hyperbaric oxygen therapy; NBA: normobaric air; NO: nitric oxide; NOS: nitric oxide synthase; TUNEL: terminal deoxynucleotidyl transferase (TdT)-mediated deoxyuridine triphosphate (dUTP)-biotin nick end labeling.

## Authors' contributions

CDL, IHW, TCH and MHT conceived this experiment. Animal studies were performed by CDL, MHT and TCH. Immunohistochemistry was performed by CDL, IHW, MCK and CHL. Confocal microscopy was performed by CDL, MHT and CHW. Data acquisition, analysis and manuscript preparation were performed by CDL, IHW, MCK, MHT and CHL. The manuscript was finally edited by CDL, CHL, MCK and CHW. All authors had final approval over both the submitted and published versions.
